# The Influence of the Environment and Clothing on Human Exposure to Ultraviolet Light

**DOI:** 10.1371/journal.pone.0124758

**Published:** 2015-04-29

**Authors:** Jin Liu, Wei Zhang

**Affiliations:** Beijing Obstetrics & Gynecology Hospital, Capital Medical University, Beijing, China; San Gallicano Dermatologic Institute, ITALY

## Abstract

**Objection:**

The aim of this study is to determine the effect of clothing and the environment on human exposure to ultraviolet light.

**Methods:**

The ultraviolet (ultraviolet A and ultraviolet B) light intensity was measured, and air quality parameters were recorded in 2014 in Beijing, China. Three types of clothing (white polyester cloth, pure cotton white T-shirt, and pure cotton black T-shirt) were individually placed on a mannequin. The ultraviolet (ultraviolet A and ultraviolet B) light intensities were measured above and beneath each article of clothing, and the percentage of ultraviolet light transmission through the clothing was calculated.

**Results:**

(1) The ultraviolet light transmission was significantly higher through white cloth than through black cloth; the transmission was significantly higher through polyester cloth than through cotton. (2) The weather significantly influenced ultraviolet light transmission through white polyester cloth; transmission was highest on clear days and lowest on overcast days (ultraviolet A: *P*=0.000; ultraviolet B: *P*=0.008). (3) Air quality parameters (air quality index and particulate matter 2.5 and 10) were inversely related to the ultraviolet light intensity that reached the earth’s surface. Ultraviolet B transmission through white polyester cloth was greater under conditions of low air pollution compared with high air pollution.

**Conclusion:**

Clothing color and material and different types of weather affected ultraviolet light transmission; for one particular cloth, the transmission decreased with increasing air pollution.

## Introduction

Ultraviolet light refers to electromagnetic radiation at wavelengths between the visible and X ray spectrums. It can be divided into ultraviolet A (UVA, 315–400 nm), ultraviolet B (UVB, 280–315 nm) and ultraviolet C (UVC, 100–280 nm) [1] based on wavelength. When sunlight travels through the atmosphere, all of the UVC and 90% of the UVB are absorbed by ozone and oxygen, but the atmosphere has little influence on UVA. Therefore, the ultraviolet light that eventually reaches the ground is predominantly UVA, with a small percentage of UVB [2].

Ultraviolet light is closely associated with human health. The ultraviolet light in sunlight can cause both acute and chronic damage to the skin, eyes and immune system. Globally, approximately 60,000 persons are dying from diseases caused by ultraviolet light irradiation, predominantly malignant melanoma [3]. However, 7-dehydrocholesterol in the skin is converted into vitamin D through multiple processes after exposure to UVB, and vitamin D subsequently modulates calcium metabolism in the body, which is essential for the metabolism of numerous cell types and for bone health. The composite vitamin D irradiated by sunlight is the major source of vitamin D in the human body; dietary vitamin D accounts for only a small proportion (10%) [4]. Insufficient sunlight exposure can eventually result in vitamin D deficiency, resulting in improper calcium homeostasis in the body, secondary hyperparathyroidism, osteoporosis and fragility fractures as well as infantile rickets [5]. There is evidence that many factors influence human exposure to ultraviolet light; the natural factors include latitude, season, ozone, cloud cover, aerosol, and reflectivity, and the individual factors include age, clothing, and sunscreen use. [6]. Numerous studies have analyzed the relationships between air pollution, ultraviolet light intensity and vitamin D levels in humans, and the evidence suggests a significant negative correlation between these parameters [7–9]. Clothing is a protective screen between the skin and ultraviolet light. In vitro experiments have revealed that the color, material, and tightness of clothing, in addition to humidity and other factors, affect ultraviolet light transmission [10–13], but there have been few studies on the human body. This study was performed in Beijing, one of the cities in China with the most serious air pollution. The UVA and UVB intensities were monitored at different times each day, multiple air quality indices were recorded, and the correlations between the color, material and tightness of clothing and ultraviolet light intensity and transmission were analyzed. In addition, a mannequin was used in our study to measure ultraviolet intensity at different anatomical sites. This approach avoided damage caused to a person as the research object and promoted a better understanding of the natural and individual factors that influence human exposure to ultraviolet light.

## Materials and Methods

### Ethics Statement

This research was approved by the ethics committee office of Beijing Obstetrics & Gynecology Hospital, Capital Medical University. The project was conducted in the field at Beijing Obstetrics & Gynecology Hospital to measure ultraviolet intensity. This study did not influence the surrounding environment or disturb the normal working order in the hospital. In addition, the experimenter engaged in good ultraviolet protection measures.

### Instruments and Materials

The following ultraviolet radiometers were used in this study: UV-A 297 Probe (wavelengths 275–330 mm) and UV-B 365 Probe (wavelengths 320–400 mm) (both were obtained from the Photoelectric Instrument Factory of Beijing Normal University, Beijing, China). A TM 827 hygrothermograph (thermometer and hygrometer) produced by Hong Kong-based Tecman Electronic Instrument Holdings Limited was also utilized. One of the most important tools used in this study was a mannequin representing a 12-year-old child with a height of 140 cm. Three types of clothing were used to determine differences based on color and material: pure cotton T-shirts (one white and one black) and polyester cloth (white), all size L and purchased from a local supplier. All the clothing was washed and starched ten times before the experiment.

### Methods

#### Detection of Ultraviolet Light Intensity on the Ground

Measurements were performed once per hour from 7 AM to 6 PM each day. The ultraviolet radiometer probes were placed on the ground, and the UVA and UVB intensities were recorded.

#### Detection of Ultraviolet Light Intensity on the Mannequin

The following detection protocol was performed each day at three different times (9:00, 12:30, and 16:00). (1) Ultraviolet radiometers were utilized to determine the intensities of UVA and UVB at four anatomical sites: the xiphoid process, the intersection between the angulus inferior line of the scapula and the posterior midline, the left shoulder, and the left chelidon. (2) After the three different types of clothing were placed on the mannequin, the ultraviolet radiometer probes were placed in close contact with the skin of the mannequin underneath the clothing (with the goal of eliminating any distance between the clothing and the probes), and the UVA and UVB intensities were then measured at the abovementioned four positions. (3) The white T-shirt and the white polyester clothing were subsequently placed on the mannequin, and the ultraviolet radiometer probes were placed on the mannequin’s skin at a distance of 1 cm from the clothing; the UVA and UVB intensities at the abovementioned four positions were measured. (4) Because humans are constantly moving when they are outside, measurements were taken with the mannequin facing east, south, west and north at various times, and the ultraviolet light intensity absorbed by each piece of clothing and anatomical site was averaged. (5) The percentage of ultraviolet light transmission was calculated using the following equation: ultraviolet light transmission = ultraviolet light intensity with clothing as a screen / ultraviolet light intensity with direct exposure to sunlight (%). (6) The ultraviolet transmission ratio was calculated using the following equation: ultraviolet transmission ratio = ultraviolet transmission of clothing off skin with 1-cm gaps / ultraviolet transmission of clothing covering the skin with no gaps.

#### Environmental Experiment

The environmental experiment was performed in Chaoyang District, Beijing, at latitude 39.9 north during June, July, and August of 2014, with solar elevation angles of 9.71°-73.60°. The mannequin was placed on open ground without shelter. The weather conditions were recorded each day as sunny (no or very few clouds), cloudy or overcast (indirect or weak solar radiation), a TM827 hygrothermograph was used to determine the ambient temperature and relative air humidity, and the Chaoyang District air quality reports published by the Environment Protection Commission were recorded in real-time. The frequently detected measures include the air quality index, PM (particular matter) 2.5, PM10, SO2, NO2, and O3. PM2.5 refers to fine particulate matter in the ambient environment with an aerodynamic diameter less than or equal to 2.5 micrometers. The air quality index, AQI, is a dimensionless index composed of PM10, PM2.5, SO2, NO2 and O3. Air quality can be divided into 5 grades based on the AQI: Excellent (0–50), Good (50–100), Mild Pollution (100–150), Moderate Pollution (150–200) and Serious Pollution (200–300).

### Statistical Analysis

The data entry and analysis were performed using Excel 2007 and SPSS16.0, respectively. The ultraviolet light intensity and transmission through clothing are presented as the mean ± standard deviation. The transmission under various circumstances was compared with a t-test (pairwise comparison) or a variance analysis (comparison of three or more variables). If the results were significantly different, the least significant difference method (LSD method, pairwise comparison) was used. In addition, Pearson’s correlation analysis was used to analyze the relationship between air quality and ultraviolet light intensity and transmission. The differences were considered statistically significant at *P*<0.05.

## Results

**Fig 1:**
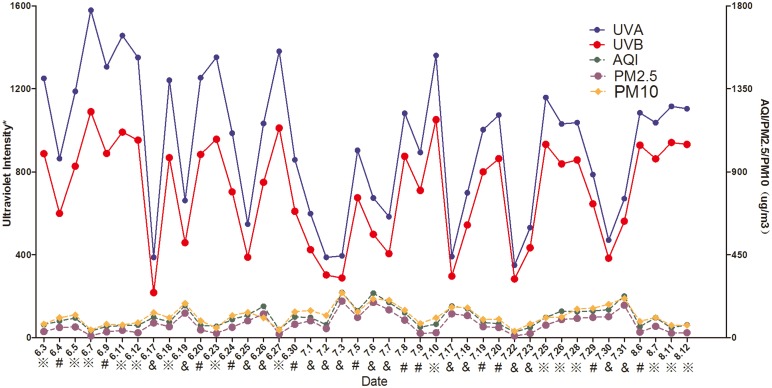
Changes in ultraviolet light intensity and air quality on all the experimental days. ※ clear day, # cloudy, and & overcast. * The UVA and UVB data are presented in μW/cm^2^ and 10×μW/cm^2^, respectively.

Data were collected on 42 days: 15 sunny days, 12 cloudy days and 15 overcast days. The ambient temperature during the experiment was 30.27±3.70°, and the relative humidity was 57.80±18.20%. Fig 1 depicts curves representing the changes in ultraviolet (UVA and UVB) light intensity and air quality (AQI, PM2.5, and PM10) on all the experimental days.

### The Influence of Different Types of Clothing on Ultraviolet Light Transmission

**Fig 2:**
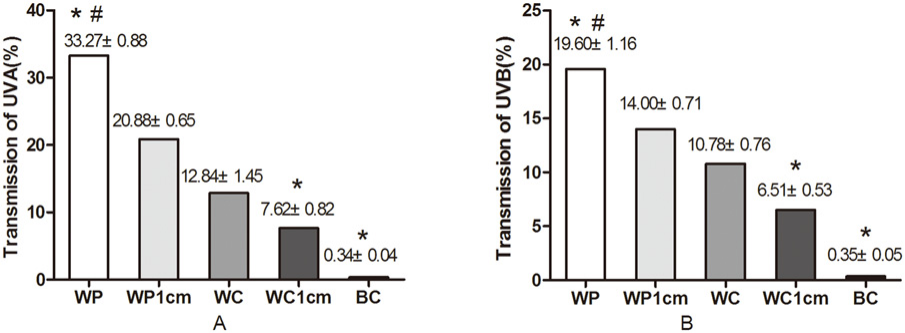
Ultraviolet light transmission through different types of clothing (%). WP, White Polyester; WC, White Cotton; BC, Black Cotton. WP1cm and WC1cm indicate that the distance between the clothing and the skin was 1 centimeter. * The transmission through this type of clothing was significantly different (*P*<0.05) from that through WC. # The transmission through WP was significantly different (*P*<0.05) from that through WP1cm.

There were tremendous differences in ultraviolet light transmission through clothing of different color, material and tightness (Fig 2). The transmission was significantly higher through white cotton than through black cotton (*P*<0.05), and the transmission was markedly higher through white polyester than through white cotton (*P*<0.05). Ultraviolet light transmission was noticeably higher when the white polyester clothing covered the skin with no gaps than when it was 1 cm from the skin (*P*<0.05); similar findings were obtained for white cotton clothing. In addition, the ultraviolet transmission ratio of clothing off skin with 1-cm gaps to the clothing covering the skin with no gaps was calculated. The polyester clothing ratio of UVA transmission was 0.62 and that of UVB was 0.71. The cotton clothing ratio of UVA was 0.59 and 0.60 for UVB.

### The Influence of Different Weather Conditions on Ultraviolet Light Intensity and Transmission

**Fig 3:**
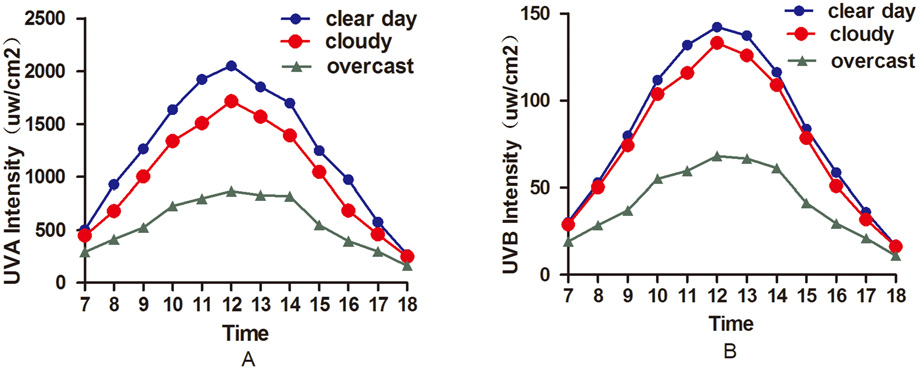
Changes in ultraviolet light intensity at different times and in three types of weather. Regardless of the weather, the UVA and UVB intensities were highest at 12:00 each day. There were significant differences in the UVA intensity among the three types of weather; the UVA and UVB intensities peaked on clear days and were lowest on overcast days.

**Fig 4:**
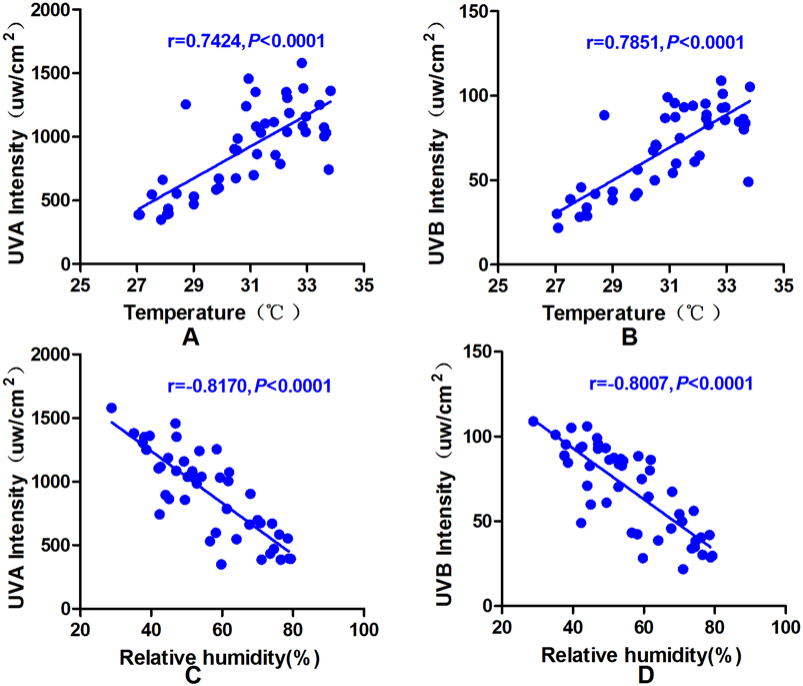
The relationship between ultraviolet intensity and humidity and temperature. A/B: Pearson’s correlation analysis revealed a significant positive correlation between temperature and UVA/UVB intensity (UVA: r = 0.7424, *P*<0.0001; UVB: r = 0.7851, *P*<0.0001). C/D: Pearson’s correlation analysis showed a significant negative correlation between relative humidity and UVA/UVB intensity (UVA: r = -0.8170, *P*<0.0001; UVB: r = -0.8007, *P*<0.0001).

There were significant differences in the ultraviolet light intensity on sunny, cloudy and overcast days (UVA: *P* = 0.000; UVB: *P* = 0.000). The ultraviolet light intensity was highest on sunny days, followed by cloudy and overcast days. Table 1 and Fig 3 present the changes in the ultraviolet light intensity at different times based on the three weather conditions. In addition, there was a positive correlation between ultraviolet intensity and temperature (UVA:*P*<0.0001, r = 0.7424; UVB: *P*<0.0001, r = 0.7851), and dramatic negative correlations were observed between ultraviolet intensity and relative air humidity (UVA:*P*<0.0001, r = -0.8170; UVB: *P*<0.0001, r = -0.8007) (Fig 4).

**Table 1:** Ultraviolet light intensity at different times in three types of weather (μW/cm^2^).

	UVA			UVB		
	Clear	Cloudy	Overcast	Clear	Cloudy	Overcast
7:00	502.93±132.68	447.25±100.16	292.63±121.48	33.90±58.80	28.40±6.36	18.50±7.86
8:00	929.07±206.23	679.50±174.76	414.18±204.56	61.51±10.80	49.33±10.57	28.49±12.60
9:00	1263.93±193.30	1005.54±192.99	524.03±259.59	91.01±11.65	71.34±12.82	37.59±17.66
10:00	1636.47±290.86	1337.50±220.13	727.40±309.66	125.15±16.67	100.39±14.49	52.75±22.63
11:00	1921.40±265.42	1508.42±470.54	796.73±413.61	147.16±16.77	111.42±31.91.	56.87±30.59
12:00	2051.57±271.90	1715.21±416.68	867.14±426.94	156.78±14.36	128.31±31.45	62.89±31.09
13:00	1851.43±286.24	1569.04±368.87	830.46±294.22	148.06±18.03	124.45±29.42	67.70±24.06
14:00	1699.82±257.78	1392.79±262.13	820.07±323.47	130.19±13.44	108.90±18.49	62.70±26.69
15:00	1248.68±219.58	1047.50±308.89	547.70±172.50	92.38±14.55	79.13±23.98	41.67±13.21
16:00	974.29±192.57	683.71±209.90	395.00±198.18	70.08±11.69	51.72±14.78	29.95±15.13
17:00	575.54±100.01	458.88±110.95	297.65±130.37	40.13±5.89	31.03±6.44	21.27±95.54
18:00	268.61±56.84	248.25±60.53	162.54±60.91	17.41±2.96	15.96±4.08	11.16±49.29

**Fig 5:**
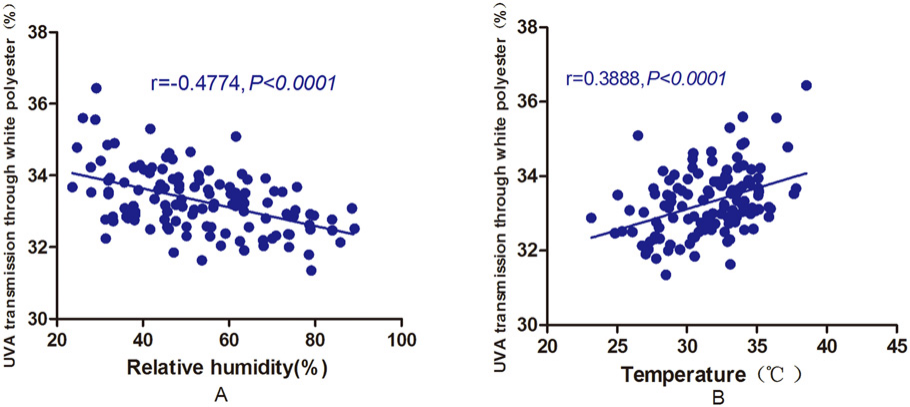
The relationship between environmental relative humidity and temperature and UVA transmission through white polyester. A: Pearson’s correlation analysis revealed a significant negative correlation between relative humidity and UVA transmission through white polyester (r = -0.4774, *P*<0.0001). B: Pearson’s correlation analysis showed a significant positive correlation between temperature and UVA transmission through white polyester (r = 0.3888, *P*<0.0001).

UVA and UVB transmission was significantly different through white polyester and white cotton under the sunny, cloudy and overcast conditions (sunny days > cloudy days > overcast days); however, there was no significant difference in the transmission through other types of clothing (Table 2). Moreover, there was a positive correlation between UVA and UVB transmission through white polyester and the temperature and a dramatic negative correlation was observed between UVA and UVB transmission and relative humidity (i.e., higher temperature correlated with lower relative humidity and higher transmission; Fig 5).

**Table 2:** Ultraviolet light transmission through clothing in different types of weather (%).

	UVA				UVB			
	Clear	Cloudy	Overcast	*P*	Clear	Cloudy	Overcast	*P*
WP	33.86±0.81	33.23±0.77	32.72±0.65	*P* = 0.000^#^	20.14±1.23	19.63±0.97	19.03±0.96	*P* = 0.000^#^
WC	12.84±1.79	12.58±1.33	13.05±1.10	*P* = 0.340	11.05±0.85	10.67±0.69	10.60±0.66	*P* = 0.012^#^
BC	0.34±0.04	0.33±0.03	0.34±0.04	*P* = 0.532	0.36±0.06	0.35±0.04	0.36±0.05	*P* = 0.587

WP, White Polyester; WC, White Cotton; BC, Black Cotton. WP1cm and WC1cm indicate that the distance between the clothing and the skin was 1 centimeter.

^#^ There were significant differences in UVA transmission among the three types of weather (*P*<0.05); the intensities of both UVA and UVB peaked on clear days and were lowest on overcast days.

### The Influence of Air Quality on Ultraviolet Light Intensity and Transmission

**Fig 6:**
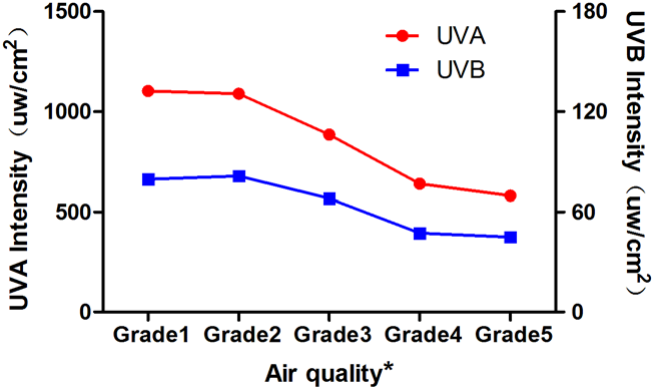
Ultraviolet intensity in 5 air quality grades. * The air quality was divided into five grades based on the AQI: grade 1, 0–50, Excellent; grade 2, 51–100, Good; grade 3, 101–150, Mild Pollution; grade 4, 151–200, Moderate Pollution; grade 5, 201–300, Serious Pollution. As air pollution increased, the ultraviolet intensity decreased.

**Fig 7:**
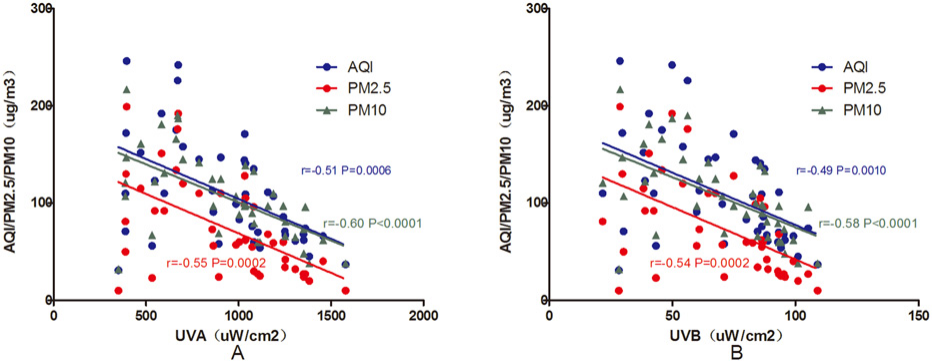
The relationship between UVA and UVB intensity and air quality parameters (AQI, PM2.5, and PM10). A: Pearson’s correlation analysis demonstrated a significant negative correlation between UVA intensity and air quality parameters (AQI: r = -0.51, *P* = 0.0006; PM2.5: r = -0.55, *P* = 0.0002; and PM10: r = -0.60, *P*<0.0001). B: Pearson’s correlation analysis revealed a significant negative correlation between UVB intensity and air quality parameters (AQI: r = -0.49, *P* = 0.0010; PM2.5: r = -0.54, *P* = 0.0002; and PM10: r = -0.58, *P*<0.0001).

As previously mentioned, air quality is divided into 5 grades. Over the 42 days, “Excellent” occurred on only 3 days, “Good” on 18 days, “Mild pollution” on 12 days, “Moderate Pollution” on 6 days, and “Serious Pollution” on 3 days. On “Excellent” air quality days, UVA and UVB intensities were 1103.53±660.13 μW/cm^2^ and 79.45±44.44 μW/cm^2^, respectively, whereas on “Serious Pollution” days, UVA and UVB intensities were 579.86±160.10 μW/cm^2^ and 44.95±14.36 μW/cm^2^, respectively (Fig 6). As air pollution increased, the ultraviolet intensity decreased. There was a slight negative correlation between air quality and ultraviolet light intensity. Pearson’s correlation analysis of the relationship between the average ultraviolet intensity per day and air quality (AQI, PM2.5, PM10, SO_2_, NO_2_, and O_3_) revealed negative correlations between AQI, PM2.5, and PM10 and UVA and UVB intensity (Fig 7).

**Fig 8:**
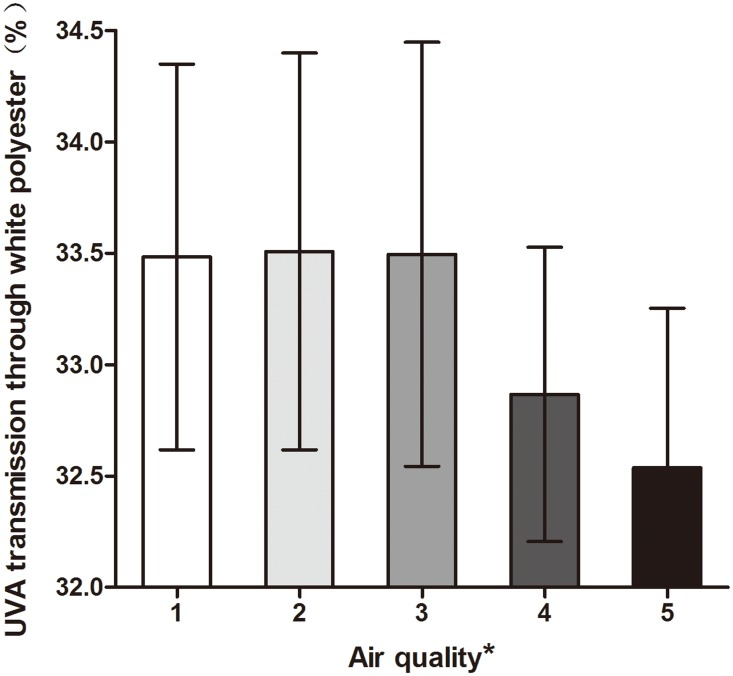
UVA transmission through white polyester with different levels of air pollution. The data on the Y-axis begin at 32%. * The air quality was divided into five levels based on the AQI: 1: 0–50, good; 2: 51–100, moderate; 3:101–150, unhealthy for sensitive groups; 4: 151–200, unhealthy; 5: 201–300, very unhealthy. UVA transmission in the presence of air quality levels 1, 2, and 3 was significantly different from that for levels 4 and 5.

Changes in air quality heavily influenced UVA transmission through white polyester clothing (Fig 8). In this experiment, the ultraviolet light transmission data were divided into 5 groups based on the AQI (Excellent, Good, Mild Pollution, Moderate Pollution and Serious Pollution), and the ultraviolet light transmission was compared. There was a clear difference in UVA transmission through white polyester in the different air quality categories (*P* = 0.003). The differences were statistically significant between most of the air quality categories: Excellent (33.48±0.87%, *P* = 0.034), Good (33.51±0.89%, *P* = 0.022), Mild (33.50±0.95%, *P* = 0.028) and Moderate (32.87±0.66%). The UVA transmission in the Excellent, Good and Mild Pollution categories was also significantly different from that in the Serious Pollution category (32.54±0.71%; *P* = 0.002, 0.001, and 0.002, respectively). However, there were no obvious differences in the transmission through other types of clothing based on air quality category. The correlations between air quality (six indices, including AQI and PM2.5) and ultraviolet light transmission were analyzed; UVA transmission through white polyester was negatively correlated with AQI and PM2.5 (AQI: *P*<0.0001, r = -0.3606; PM2.5: *P*<0.0001, r = -0.3707).

### Influence of Different Anatomical Sites on Ultraviolet Light Intensity

**Fig 9:**
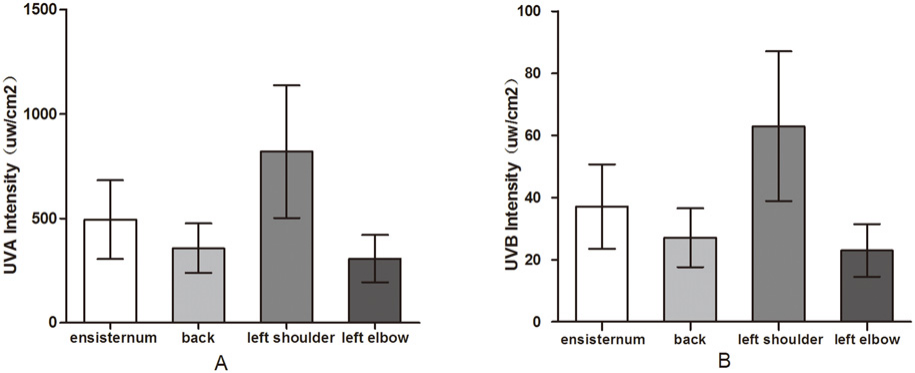
Ultraviolet light intensity at different anatomical sites. Although the back and the left elbow showed no significant differences in exposure to ultraviolet light, there were significant differences in the UVA and UVB intensities experienced at the four anatomical sites.

There were marked differences in the ultraviolet light intensity experienced at the four anatomical sites (Fig 9). The intensities at the xiphoid process, back, left shoulder and left elbow were 490.52±186.75 μW/cm^2^, 354.58±117.52 μW/cm^2^, 814.43±315.74 μW/cm^2^, and 304.56±113.46 μW/cm^2^, respectively. The intensities at the back and the left elbow were not significantly different (UVA: *P* = 0.229; UVB: *P* = 0.186), but the intensities at the xiphoid process and the left shoulder were significantly different (*P*<0.0001). The sequence of ultraviolet intensity exposure was left shoulder > xiphoid process > left elbow = back.

## Discussion

Ultraviolet light has an important environmental influence on human health; therefore, it is necessary to increase our knowledge of changes in ultraviolet light intensity. Gao et al. [14] analyzed the daily and seasonal changes in ultraviolet light in Shenyang, China, between 2006 and 2009 and found that the daily UVA and UVB intensities formed a unimodal curve; the intensity was strongest at noon, with UVA and UVB intensities of 25–30 W/m^2^ and 0.6–0.7 W/m^2^, respectively, in the summer. The ultraviolet intensities during the four seasons are as follows: summer > spring > autumn > winter. In this study, the daily changes in ultraviolet light intensity were identical to those mentioned above, and the data formed a unimodal curve. UVB plays an essential role in the production of vitamin D in humans, and UVB-irradiated vitamin D satisfies most of the human biological need. A study of American children [15] discovered that the seasonal body levels of vitamin D ranged as follows: summer > spring > autumn > winter, which obeys the laws governing UVB intensity. Ultraviolet radiation is stronger in the summer and thus more easily damages the skin and eyes; therefore, protective measures should be taken. In winter, ultraviolet radiation is weaker, and heavy clothing is necessary; under these conditions, humans are exposed to less UVB, thus leading to vitamin D deficiency. Consequently, outdoor activity during winter should increase on sunny days with good air quality, and vitamin D consumption through an adequate diet should also increase to prevent deficiencies. Every part of the human body can receive ultraviolet light, whether on the horizontal, vertical or inclined planes; the differences in the receiving angle impact the intensity of the ultraviolet irradiation. In this experiment, a mannequin was used to determine the intensity of ultraviolet irradiation at multiple anatomical sites, and the results indicated that the shoulder received the greatest average daily amount of ultraviolet light, followed by the xiphoid process, the left elbow and the back. Hence, in daily life, it is important to better protect the body parts (shoulders and front chest) that receive the most exposure to ultraviolet light.

Clothing is the most effective means of protecting skin from the damage caused by ultraviolet light. Ultraviolet light transmission differs based on the color and material of the clothing as well as its distance from the skin. First, washing influences ultraviolet light transmission through the clothing. As observed with a light microscope, the size of the holes between the fibers can be reduced from 8% to 3.9%, and ultraviolet light transmission is thereby decreased [10]. Prior to this experiment, the utilized clothing was washed ten times with water to more accurately resemble the clothing people wear daily and to avoid the influence of washing on ultraviolet light intensity and transmission. Second, differences in clothing fibers influence ultraviolet light transmission. In this study, the white polyester cloth allowed approximately 33.27% of UVA and 19.60% of UVB to pass through, and the transmission was significantly lower through white cotton than through white polyester. Another study [16] also found that UVB transmission was considerably higher through polyester clothing than cotton clothing (2.4% vs. 0.8%). However, the data in this previous study differ from our findings, potentially due to differences in other properties, such as the thickness and density, of the selected clothing. For this reason, assuming that the color and other properties are similar, cotton clothing should be worn in summer to enhance sun protection. Third, the color of the clothing influences ultraviolet light transmission. This study demonstrated that ultraviolet light transmission was dramatically higher through white cotton than black cotton. Black items absorb most light, including infrared, visible and ultraviolet light; infrared and visible light contain larger amounts of heat energy. When sunlight is absorbed by black clothing, the light energy is converted into heat and the temperature rises, causing the person to feel hotter when wearing black clothing in the summer. In addition, black clothing absorbs most ultraviolet rays, which decreases the amount of ultraviolet light that reaches the skin; thus, black clothing is a more effective sunscreen. Fourth, the distance from the skin influences ultraviolet light transmission. According to one study [17], clothing on the skin enabled a lower Ultraviolet Protection Factor (UPF) compared with clothing off the skin (2–4 mm from the skin). Moreover, the ratio of UPF on skin to off skin ranged from 0.37 to 1 for cotton and polyester clothing. In other words, ultraviolet transmission of clothing on skin was higher than that of clothing off skin (2–4 mm from the skin), and the ultraviolet transmission ratio of clothing off the skin to clothing on skin ranged 0.37 to 1. The paper considered that in “off skin” testing, ultraviolet light scattered and diffused after passing through the spaces between the yarns of the clothing, thereby reducing ultraviolet intensity. Our study compared the ultraviolet light transmission through two types of clothing either covering the skin with no gap or at a distance of 1 cm, and the data were similar to the above study. The ratio ranged from 0.59 to 0.71. Thus, a potentially effective way to block the sun during the summer is to wear loose clothing. In conclusion, clothing color, material and the distance from the skin can influence ultraviolet transmission. When two of the three factors are held constant, a change in the third factor influences ultraviolet transmission. These results may guide ultraviolet protection and decrease ultraviolet transmission. Furthermore, the fiber structure, tightness of the weave [18], and use of brightening agents also affect ultraviolet light transmission.

The natural factors that influence ultraviolet light intensity include solar altitude, ozone, cloud cover, air quality and pollution, reflectivity, and cloud height, thickness and spatial distribution; all of these factors impact the intensity of the ultraviolet light that reaches the ground. The ultraviolet light intensity on the ground is less affected on sunny days without cloud cover than on overcast days with thick layers of cloud cover [6]. This study demonstrated that the ultraviolet light intensity was greater on sunny days than on overcast days, as was the transmission through white polyester clothing. Overall, white polyester clothing enables more serious damage to the skin than cotton clothing on sunny days. We were unable to identify any literature establishing a connection between air humidity and ultraviolet light transmission through clothing. This experiment identified a negative correlation between humidity and transmission through white polyester clothing that is likely attributable to the relationship between humidity and weather conditions. Normally, humidity is lower on sunny days than on cloudy days, and our experimental data suggested that ultraviolet light transmission was higher on sunny days than on cloudy days. These results suggested a connection between humidity and ultraviolet light transmission through white polyester clothing, but humidity does not influence transmission; importantly, both humidity and ultraviolet light transmission are associated with air conditions. The correlation between temperature and ultraviolet light transmission through clothing was similar. In this experiment, the ultraviolet light detection point was a lawn with approximately 5% [6] reflectivity, thereby preventing the influence of excessive ground reflectivity on ultraviolet light intensity. The experiment was conducted in Beijing, one of the cities with the most serious pollution; the obvious changes in air quality enabled the collection of powerful data for analyzing the correlation between air pollution and ultraviolet light intensity. The standard air pollutants, based on human health and potential environmental hazards, are divided into 6 types: O_3_, PM, NO_2_, SO_2_, CO and lead [19]. Recently, numerous studies have analyzed the correlations between air pollution, ultraviolet light intensity and vitamin D levels. Farhad et al. [8] showed that the UVB intensity was clearly lower in areas with serious air pollution than in locations with little air pollution. Roya et al. [9] performed a Pearson correlation analysis of AQI and UVB intensity and found a negative correlation between these two factors. Regarding populations with vitamin D deficiency, researchers studied women and children and observed more severe and widespread vitamin D deficiency among infants [7], children [9] and healthy women in areas with heavy air pollution compared with the same groups in areas with little air contamination because of reduced UVB intensity. In the experiment reported herein, 6 air quality measures were selected (PM2.5, PM10, NO_2_, SO_2_, O_3_ and AQI), and a notable negative correlation was observed between AQI, PM2.5 and PM10 and UVA and UVB intensity. As air pollution increased, the ultraviolet intensity decreased. This finding may be related to ultraviolet light diffusion through aerosols. When the air pollution is serious, there are more aerosols in the air, and more ultraviolet light is diffused. A recent paper [20] reported that air quality was related to many factors, including air humidity, rainfall, and wind speed. These authors found that the AQI and relative humidity were positively correlated. Increased relative humidity can inhibit airborne particulate diffusion and result in major particulate accumulation. In our study, we found that the ultraviolet intensity and relative humidity were negatively correlated. However, the influence of humidity on ultraviolet intensity due to its influence on air quality is unknown. The interrelationship of the three factors requires further investigation. The previous study [20] also mentioned that other meteorological conditions can influence air quality, such as wind speed and rainfall. We did not record these parameters in the current study. In addition, white polyester clothing ultraviolet transmission was significantly decreased with increasing air pollution index. However, it is unclear if the observed decrease in ultraviolet transmission through clothing is due to the lower ultraviolet intensity caused by the increased air pollution index.

People tend to prefer indoor activities on days with heavy pollution; together with the relatively weaker ultraviolet light intensity, these factors result in less UVB absorption through the skin and consequently a lack of vitamin D. People living in heavily polluted cities should consume foods that contain more vitamin D to avoid becoming vitamin D deficient. Prolonging one’s sun exposure is another option. This study demonstrated that white polyester clothing enabled greater UVA transmission when the air quality was excellent than under conditions of moderate or severe pollution, potentially because of the larger fiber gaps in polyester clothing. Contaminating particulate can greatly impact ultraviolet light transmission, but the impact on cotton was relatively modest because of its tight fiber structure. The severity of the air pollution was negatively correlated with ultraviolet light intensity, and air quality influenced ultraviolet light intensity and transmission through clothing.

## Conclusion

Our results suggest that ultraviolet intensity and transmission can be influenced by clothing color, material and the distance from the skin. Ultraviolet transmission through clothing covering the skin with no gaps is higher than through clothing covering the skin with 1-cm gaps for both cotton and polyester clothing. Ultraviolet intensity and transmission can be influenced by weather conditions and air quality. Ultraviolet intensity on a sunny day is significantly higher than on cloudy and overcast days, as is the transmission for certain clothing such as white cotton and white polyester clothing. Furthermore, ultraviolet intensity is negatively correlated with air quality indexes, as is the ultraviolet transmission for certain clothing such as white polyester. However, more research is needed to better understand the impact of air quality on ultraviolet light transmission.

## Supporting Information

S1 File Editorial Certificate. The manuscript was edited by highly qualified native English speaking editors at American Journal Experts, and this certificate was obtained.
(PDF)

S2 File Ethical Approval Form. This research was approved by the ethics committee office of Beijing Obstetrics & Gynecology Hospital, Capital Medical University.
(PDF)
