# Supplementary material for: The Influence of the Environment and Clothing on Human Exposure to Ultraviolet Light
Source: PLoS One. 2015 Apr 29;10(4):e0124758. doi: 10.1371/journal.pone.0124758 (PMC4414538; doi:10.1371/journal.pone.0124758)
Supplement: S2 File — This research was approved by the ethics committee office of Beijing Obstetrics & Gynecology Hospital, Capital Medical University. (PDF) [file pone.0124758.s002.pdf]

BEIJING OBSTETRICS & GYNECOLOGY HOSPITAL, CAPITAL MEDICAL UNIVERSITY

Ethical Approval Form

<table>
<tr><td rowspan="6">General information on the project</td><td>Protocol name</td><td colspan="3">The Influence of the Environment and Clothing on Human Exposure to Ultraviolet Light</td></tr>
<tr><td>Protocol type</td><td colspan="3">Scientific research</td></tr>
<tr><td rowspan="2">Overall responsibility</td><td rowspan="2">☑ Yes
☐ No</td><td rowspan="2">Multi-center</td><td>☑ No</td></tr>
<tr><td>☐ Yes; continue to complete: ☐ International ☐ Domestic</td></tr>
<tr><td>Type of review</td><td colspan="3">☑ Initial review ☐ Tracking review</td></tr>
<tr><td>Brief description of project</td><td colspan="3">This project is carried in a field at Beijing Obstetrics & Gynecology Hospital to measure ultraviolet intensity. This can not influence surrounding environment and disturb normal working order in hospital. In addition, experimenter takes good ultraviolet protection measures. A mannequin is utilized to measure ultraviolet intensity at different anatomical sites and this avoids kinds of damages caused by letting a person as the research object.</td></tr>
<tr><td rowspan="5">Basic information on principal investigator of the project</td><td>Name</td><td>Wei Zhang</td><td>Department</td><td>Neonatal Intensive Care Unit, Beijing Obstetrics & Gynecology Hospital</td></tr>
<tr><td>Technical Post</td><td>Chief physician</td><td>Mobile phone</td><td>13901317623</td></tr>
<tr><td>Title</td><td>Director of department</td><td>E-mail</td><td>zw2966@sina.com</td></tr>
<tr><td>Correspondence Add.</td><td colspan="3">Neonatal Intensive Care Unit, Beijing Obstetrics & Gynecology Hospital, No. 251, Yaojiayuan Road, Changyang District, Beijing</td></tr>
<tr><td>Main research direction</td><td colspan="3">Vitamin D; Ultraviolet B; Neonatal diseases</td></tr>
<tr><td rowspan="2">Review opinion of Ethics Committee office of the hospital</td><td>Project related data are complete</td><td colspan="3">☑ Yes
☐ No</td></tr>
<tr><td colspan="4">Recommendation opinion from ethical review:
This project is approved on the aspect of ethics.
Signed by the Director of Ethics Committee office of the hospital:
Teng Hong-hong
Teng Hong-hong
Date: 2014.4.15</td></tr>
</table>
